# Infliximab versus intravenous immunoglobulin for refractory Kawasaki disease: a phase 3, randomized, open-label, active-controlled, parallel-group, multicenter trial

**DOI:** 10.1038/s41598-017-18387-7

**Published:** 2018-01-31

**Authors:** Masaaki Mori, Takuma Hara, Masako Kikuchi, Hiroyuki Shimizu, Tomoyuki Miyamoto, Satoru Iwashima, Tatsuya Oonishi, Kunio Hashimoto, Norimoto Kobayashi, Kenji Waki, Yasuo Suzuki, Yoshikazu Otsubo, Hiroshi Yamada, Chikao Ishikawa, Taichi Kato, Shigeto Fuse

**Affiliations:** 1 Yokohama City University Medical Center, Yokohama, Japan; 2 Department of Pediatrics, Yokohama City University Hospital, Yokohama, Japan; 3 Children’s Medical Center, Yokohama City University Medical Center, Yokohama, Japan; 4 Department of Pediatrics, Yokosuka General Hospital Uwamachi, Yokosuka, Japan; 5 Hamamatsu University School of Medicine, Hamamatsu, Japan; 6 Department of Pediatrics, National Hospital Organization Shikoku Medical Center for Children and Adults, Zentsuji, Japan; 7 Department of Pediatrics, Nagasaki University Graduate School of Biomedical Sciences, Nagasaki, Japan; 8 Department of Pediatrics, Shinsyu University School of Medicine, Matsumoto, Japan; 9 Department of Pediatrics, Kurashiki Central Hospital, Kurashiki, Japan; 10 Department of Pediatrics, Yamaguchi University Graduate School of Medicine, Ube, Japan; 11 Department of Pediatrics, Sasebo City General Hospital, Sasebo, Japan; 12 Mitsubishi Tanabe Pharma Corporation, Tokyo, Japan; 13 Department of Pediatrics, Nagoya University Hospital, Nagoya, Japan; 14 Department of Pediatrics, NTT Sapporo Medical Center, Sapporo, Japan; 15 Present Address: Department of Lifetime Clinical Immunology, Tokyo Medical and Dental University, Tokyo, Japan; 16 Present Address: Department of Pediatrics, Hara Children’s Clinic, Tokorozawa, Japan; 17 Present Address: Department of Pediatrics, Chutoen General Medical Center, Kakegawa, Japan

## Abstract

We compared the efficacy and safety of infliximab with intravenous immunoglobulin (IVIG), a standard therapy, in a phase 3 trial (NCT01596335) for Japanese patients with Kawasaki disease (KD) showing persistent fever after initial IVIG. Patients with **i**nitial IVIG-refractory KD, aged 1–10 years, received a single dose of IV infliximab 5 mg/kg or IV polyethylene glycol-treated human immunoglobulin (VGIH) 2 g/kg on day 0. Primary outcome was defervescence rate within 48 h after the start of treatment. Safety was evaluated through day 56. Overall, 31 patients were randomized (infliximab, n = 16; VGIH, n = 15); 31.3% and 60.0% patients discontinued due to worsening KD. Defervescence rate within 48 h was greater with infliximab (76.7%) than VGIH (37.0%) (p = 0.023), and defervescence was achieved earlier with infliximab (p = 0.0072). Coronary artery lesions occurred in 1 (6.3%) and 3 (20.0%) patients receiving infliximab and VGIH, respectively, up to day 21. Adverse events occurred in 15 (93.8%) and 15 (100.0%) patients in the infliximab and VGIH groups, respectively. No serious adverse events in the infliximab group and one in the VGIH group were observed. Infliximab improved the defervescence rate within 48 h and time to defervescence versus standard therapy, and was well tolerated in patients with IVIG-refractory KD.

## Introduction

Kawasaki disease (KD) is an acute febrile disorder predominantly affecting young children, especially those aged 0–5 years^1,2^. KD frequently causes coronary artery abnormalities and acquired heart disease in children^1,2^. Therefore, the most important goal of treatment is to prevent coronary artery lesions (CALs) by suppressing acute inflammation within 10 days of the onset of illness^3^.

Intravenous immunoglobulin (IVIG) is the initial therapy for KD^3,4^ and leads to rapid defervescence and improvement of inflammatory conditions in most patients, resulting in a lower incidence of CALs; however, approximately one-fifth of patients respond inadequately to initial IVIG therapy^5–7^. Patients with KD refractory to IVIG therapy, defined as a persistent or recrudescent fever ≥24 h or ≥36 h after an initial IVIG therapy^2,7^, are at increased risk of CALs. Treatment options for initial IVIG-refractory KD include additional IVIG, prednisolone, methylprednisolone pulse, ulinastatin, cyclosporine, methotrexate, and plasma exchange; at present, an additional dose of IVIG is the most common^4,7^ and recommended^7^ therapy. However, approximately half of patients are unresponsive to IVIG retreatment^7^, leading to the investigation of several alternative treatments, including immunomodulatory agents, cytotoxic agents^2^, and interleukin (IL)-1 blockade^8^.

Serum tumor necrosis factor-α (TNF), which is a pro-inflammatory cytokine, is higher in patients with KD than in healthy children and adults and is higher in patients with than without CALs^9,10^. These results suggest that TNF is an important cause of severe complications in KD. Infliximab is a monoclonal antibody that specifically binds to TNF and inhibits its pro-inflammatory effects^11^. Several cases of infliximab-treated IVIG-refractory KD have been reported^2,12–16^. A phase 1, randomized, multicenter clinical trial of infliximab in initial IVIG-refractory patients with KD treated with IVIG on or before day 14 of fever reported that infliximab was well tolerated^14^. However, there have been few randomized trials in this setting and none in Japan, which has the highest incidence of KD^12^, and the usefulness of infliximab in IVIG-refractory KD is unclear. Therefore, we conducted a phase 3, randomized, open-label, active-controlled, parallel-group, multicenter trial to compare the efficacy and safety of infliximab treatment within 8 days of illness onset with an additional dose of IVIG in Japanese patients with initial IVIG-refractory KD.

## Results

**Figure 1:**
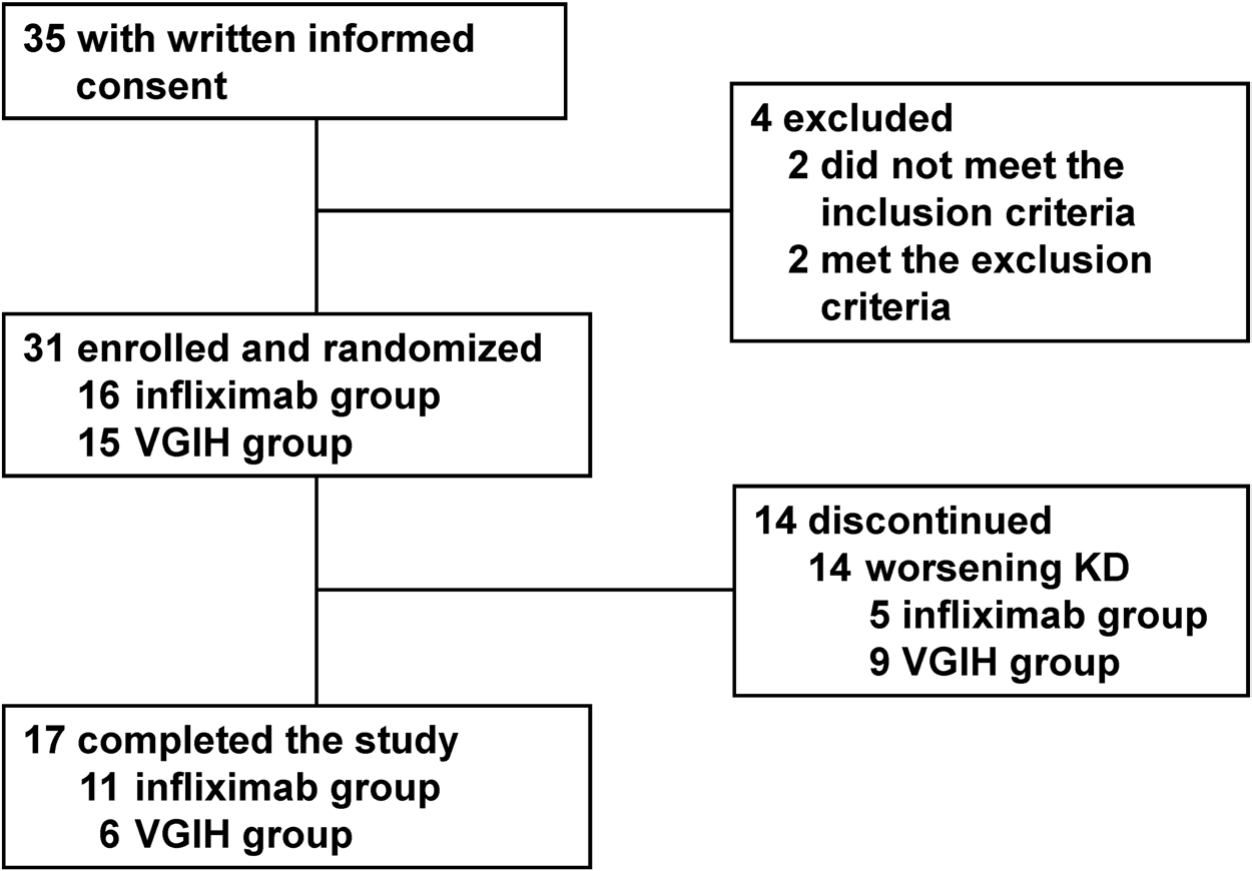
Patient disposition. KD, Kawasaki disease; VGIH, polyethylene glycol-treated human immunoglobulin.

This study started on May 2012 and ended on September 2014, but the recruitment target was not reached, owing to the very small number of patients who met the eligibility criteria. Of the 35 patients with written informed consent, 31 were enrolled and randomized (n = 16 in the infliximab group, n = 15 in the VGIH group) (Fig. 1). Five of 16 (31.3%) and nine of 15 (60.0%) patients receiving infliximab and VGIH discontinued the trial due to worsening KD (persistent fever or CALs development) and were switched to another treatment at each physician’s discretion. Patient characteristics and treatment after withdrawal from the trial are shown in Table 1.

**Table 1:** Patient characteristics.

	Infliximab (n = 16)	VGIH (n = 15)
Sex (males), n (%)	10 (62.5)	11 (73.3)
Median age at enrollment, years (range)	2.5 (1–6)	3.0 (1–4)
1 to <2, n (%)	2 (12.5)	2 (13.3)
2 to <10, n (%)	14 (87.5)	13 (86.7)
Median height, cm (IQR)	94.0 (86.5–102.5)	92.0 (89.0–96.0)
Median weight, kg (IQR)	13.75 (11.50–16.95)	13.20 (12.00–14.30)
Presence of complications, n (%)	7 (43.8)	6 (40.0)
Median duration of KD before starting treatment, days (IQR)	7.0 (6.0–7.0)	7.0 (6.0–7.0)
Major symptoms of KD, n (%)		
Fever for ≥5 days	16 (100.0)	15 (100.0)
Bilateral bulbar conjunctival congestion	15 (93.8)	15 (100.0)
Lip/oral cavity changes	16 (100.0)	15 (100.0)
Polymorphous rash	16 (100.0)	15 (100.0)
Distal extremity changes	16 (100.0)	15 (100.0)
Non-suppurative cervical lymphadenopathy	15 (93.8)	15 (100.0)
Median body temperature at enrollment, °C (IQR)	38.80 (38.40–39.75)	38.60 (37.70–39.50)
Median body temperature on day 0, °C (IQR)	39.40 (38.00–40.15)	38.80 (38.50–39.90)
Concomitant acetylsalicylic acid, n (%)	15 (93.8)	15 (100.0)
Concomitant systemic corticosteroids, n (%)	0	0
Treatment after withdrawal from the trial, n (%)	(n = 5)	(n = 9)
Immunoglobulins	3 (60.0)	3 (33.3)
Systemic corticosteroids	1 (20.0)	0
Cyclosporine	1 (20.0)	1 (11.1)
Acetylsalicylic acid	1 (20.0)	0
Infliximab (Remicade^®^)	0	5 (55.6).
Plasmapheresis	2 (40.0)	3 (33.3)

KD, Kawasaki disease; IQR, interquartile range; VGIH, polyethylene glycol-treated human immunoglobulin.

**Figure 2:**
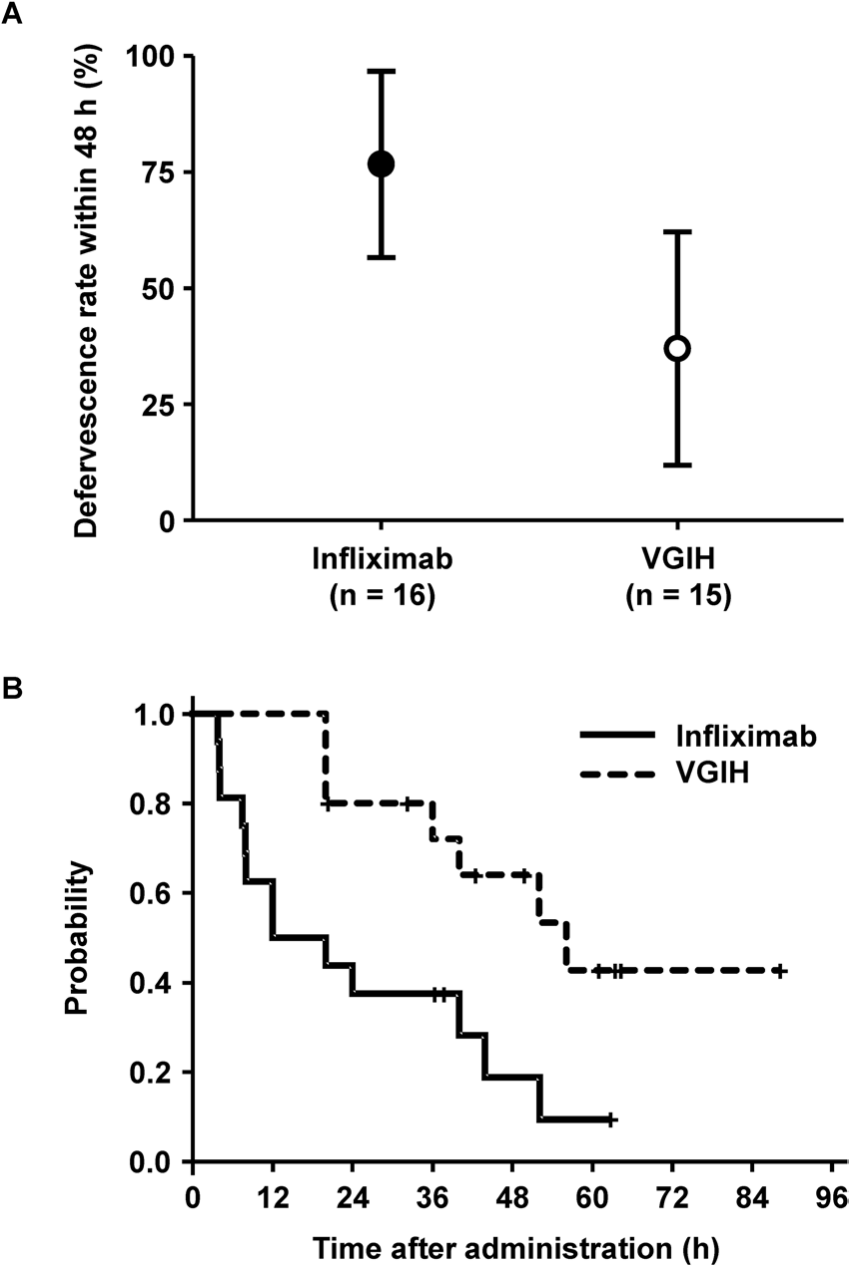
Defervescent effect. (**A**) Defervescence rate (adjusted least squares mean, 95% CI) within 48 h after study drug administration. (**B**) Kaplan–Meier plot of febrile duration. CI, confidence interval; VGIH, polyethylene glycol-treated human immunoglobulin.

The defervescence rate within 48 h was significantly greater in the infliximab group (infliximab: 76.7% [95% confidence interval: 56.6–96.7%]; VGIH: 37.0% [11.9–62.1%]; p = 0.023) (Fig. 2A). The defervescence rates at 48 h were 70.0% (7/10) and 83.3% (5/6) for males and females, respectively, in the infliximab group and were 27.3% (3/11) and 50.0% (2/4) for males and females, respectively, in the VGIH group. The median febrile period from the start of study drug administration was 16.0 h and 56.1 h in the infliximab and VGIH groups, respectively. The duration of fever was significantly shorter in the infliximab group post-hoc using the log-rank test (p = 0.0072; Fig. 2B). Axillary body temperature from 4 h to 20 h was significantly lower in the infliximab group than in the VGIH group post-hoc using an unpaired t-test (Supplementary Fig. S1).

**Figure 3:**
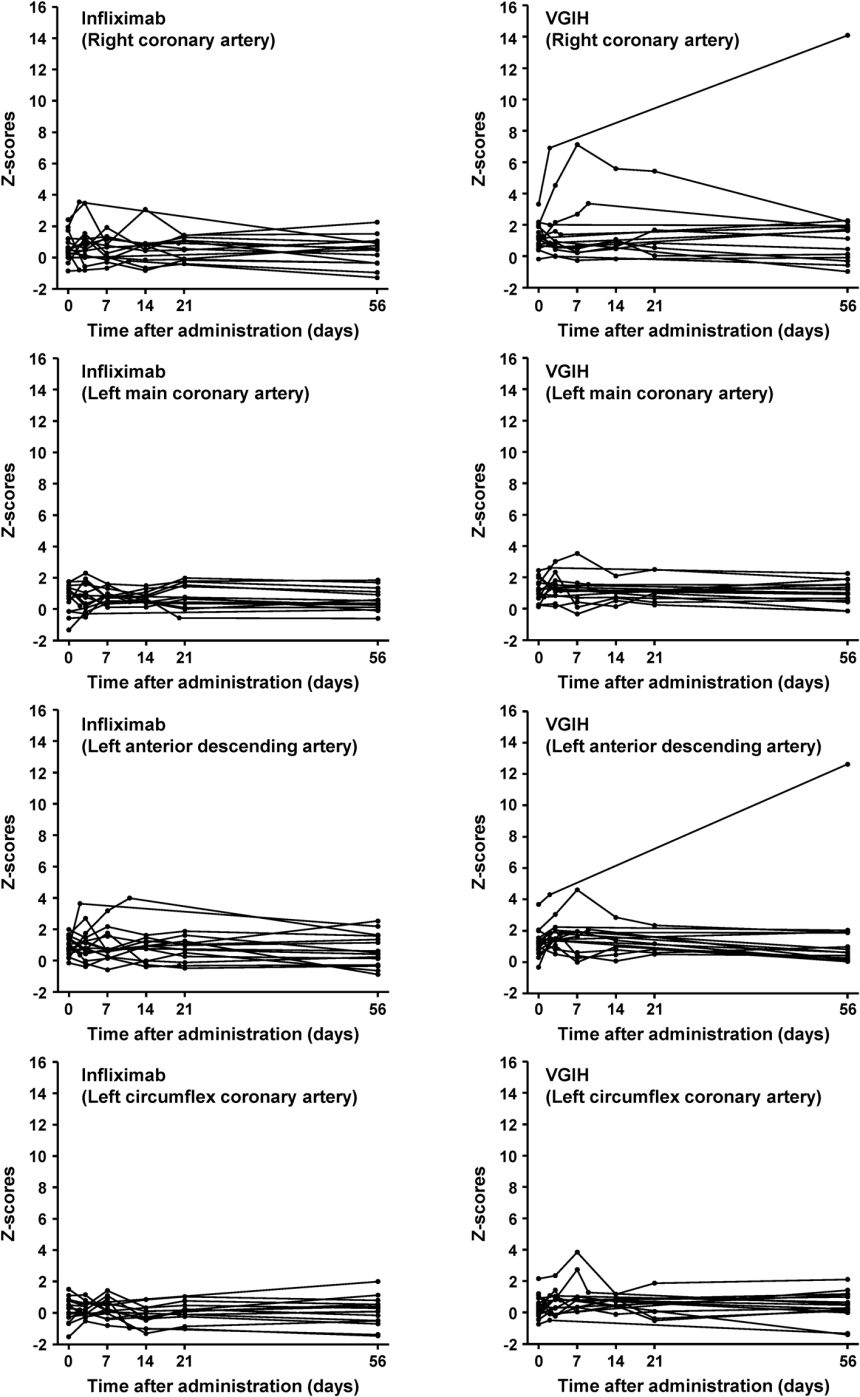
Changes in Z-scores for the internal diameter of the right coronary artery, left main coronary artery, left anterior descending artery, and left circumflex coronary artery in individual patients in each treatment group. VGIH, polyethylene glycol-treated human immunoglobulin.

CALs were found in one patient receiving infliximab (6.3%) and in three patients receiving VGIH (20.0%) through day 21. No patient had a new CAL after day 21. In the patients who were evaluated for coronary artery internal diameters (Z-score) after the start of treatment, there was no difference in the Z_max_ (largest of the right coronary artery, left main coronary artery, left anterior descending artery, and left circumflex coronary artery internal diameters) at days 0, 3, 7, 14, 21, and 56 between the infliximab group and the VGIH group post-hoc using a Mann-Whitney U test (Supplementary Fig. S2). However, two patients in the VGIH group had coronary artery Z-scores exceeding 5 (Fig. 3). In one infliximab-treated patient, lesions were found in the right coronary artery (internal diameter: 3.11 mm versus 2.42 mm on day 0; Z-score: 3.54 versus 1.93 on day 0) and left anterior descending artery (internal diameter: 3.11 mm versus 1.94 mm on day 0; Z-score: 3.63 versus 0.80 on day 0) on discontinuation day (day 2), and both lesions regressed to <3.0 mm by day 56 after plasmapheresis. A right CAL was found in three VGIH-treated patients, with internal diameters (Z-score) of 4.06 mm (6.90) on discontinuation day (day 2; persisting until day 56, even with plasmapheresis, additional IVIG, and cyclosporine treatment) versus 2.70 mm (3.31) on day 0, 3.14 mm (3.36) on discontinuation day (day 9; regressed by day 56 after treatment with additional IVIG) versus 2.09 mm (0.81) on day 0, and 5.26 mm (7.12) on trial day 7 (regressed by day 56) versus 2.52 mm (1.98) on day 0.

The improvement and resolution of clinical symptoms other than fever were comparable between treatment groups (Supplementary Fig. S3). Mean white blood cell counts were within the normal range throughout the trial in both groups (Supplementary Fig. S4). The mean neutrophil count decreased after study drug administration and was within the normal range from 1 day after infliximab administration (9777.4/μl on day 0 to 7954.9/μl on day 1), and from 3 days after VGIH administration (12,354.7/μl on day 0 to 6034.1/μl on day 3). The mean platelet count was elevated by day 7 and decreased on day 14 after infliximab (26.64, 62.19, and 45.82 × 10^4^/μl on days 0, 7, and 14, respectively) or VGIH administration (33.49, 67.31, and 51.48 × 10^4^/μl on days 0, 7, and 14, respectively). The mean albumin concentration was very low before administration of the study drugs (24.1 g/l in the infliximab group and 24.7 g/l in the VGIH group) but returned to the normal range within 14 days after infliximab (40.3 g/l on day 14) or VGIH administration (39.8 g/l on day 14). The mean C-reactive protein (CRP) concentration was above the normal range in both groups before study drug administration (8.835 mg/dl in the infliximab group and 13.764 mg/dl in the VGIH group), but it decreased from day 3 (2.577 mg/dl in the infliximab group and 5.685 mg/dl in the VGIH group) and was within the normal range by day 7 (0.807 mg/dl in the infliximab group and 0.830 mg/dl in the VGIH group), remaining almost constant until day 56.

**Figure 4:**
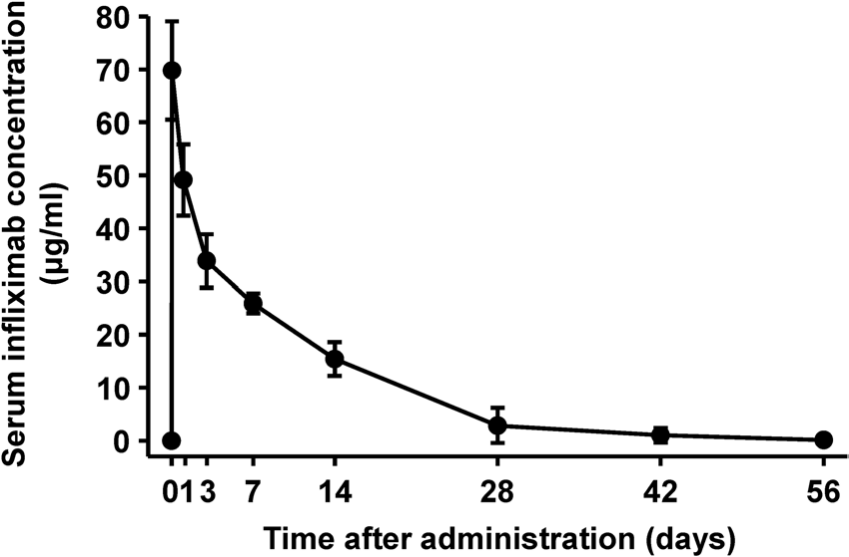
Pharmacokinetics of infliximab. Mean (standard deviation) serum infliximab concentrations after a single dose.

The mean (standard deviation) serum concentration measured 1 h after the end of infliximab infusion, time to the maximum concentration, and the elimination half-life of infliximab were 69.80 (9.26) μg/ml (Fig. 4), 3.23 (0.30) h, and 179.3 (71.2) h, respectively.

Adverse events (AEs) occurred in 15/16 (93.8%) and 15/15 (100.0%) patients in the infliximab and VGIH groups, respectively, and adverse drug reactions (ADRs) occurred in 11/16 (68.8%) and 10/15 (66.7%) (Table 2). There were no discontinuations due to AEs. There was one serious AE (relapse of KD) in a VGIH-treated patient. Infusion reactions were reported in 0/16 infliximab- and 2/15 (13.3%) VGIH-treated patients; infections were reported in 6/16 (37.5%) and 10/15 (66.7%). For infections reported in >10% of patients, we observed nasopharyngitis (4), upper respiratory tract infection (3), and upper respiratory tract inflammation (2) in the infliximab group; and nasopharyngitis (2), upper respiratory tract infection (2), bronchitis (2), and upper respiratory tract inflammation (2) in the VGIH group. The most common AE was an increase in anti–double-stranded DNA (anti-dsDNA) IgM antibodies (normal range: <6 U/ml) in the infliximab group (11/16 [68.8%]). An increase in anti-dsDNA IgG antibodies (normal range: ≤12 IU/ml) was observed in one patient (6.3%) who also showed increased anti-dsDNA IgM antibodies. Increase in anti-dsDNA IgM antibodies was the most common AE in the VGIH group (10/15 [66.7%]); however, 4/10 patients were confirmed as having an increase in anti-dsDNA IgM antibodies until day 56 after discontinuation of VGIH and subsequently infliximab (Remicade®: Janssen Biotech, Inc., PA, USA; and Mitsubishi Tanabe Pharma Corporation, Osaka, Japan). In patients with elevated anti-dsDNA antibodies, anti-dsDNA antibodies decreased to normal levels by the end of the trial and in patients who were followed after completion of the trial. Lupus-like syndrome was not observed in any patient.

**Table 2:** Safety profiles.

	Infliximab (n = 16)		VGIH (n = 15)	
	n (%)		n (%)	
AEs	15 (93.8)		15 (100.0)	
ADRs	11 (68.8)		10 (66.7)	
Serious AEs	0 (0.0)		1 (6.7)	
AEs in ≥2 patients*	**n (%)**	**n events**	**n (%)**	**n events**
Anti-dsDNA antibody increased	11 (68.8)	11	10 (66.7)	10
Epistaxis	3 (18.8)	4	4 (26.7)	7
Nasopharyngitis	3 (18.8)	3	2 (13.3)	2
Upper respiratory tract inflammation	3 (18.8)	3	2 (13.3)	2
Vomiting	1 (6.3)	2	2 (13.3)	3
Constipation	1 (6.3)	1	4 (26.7)	4
Upper respiratory tract infection	1 (6.3)	2	2 (13.3)	2
Dermatitis contact	1 (6.3)	1	3 (20.0)	3
Rash	2 (12.5)	3	0	0
Bronchitis	0	0	2 (13.3)	2
**ADRs**	**n (%)**	**n events**	**n (%)**	**n events**
Anti-dsDNA	11 (68.8)	11	10 (66.7)	10
Rash	1 (6.3)	1	0	0
Neuralgia	1 (6.3)	1	0	0

*AEs in one patient each in the infliximab group: arthropod sting, contusion, diarrhea, disuse syndrome, dry skin, ear pain, liver function test abnormal, miliaria, neck pain, neuralgia, pyrexia, increased transaminases, urticaria. AEs in one patient each in the VGIH group: activated partial thromboplastin time prolonged, anal hemorrhage, blood cholesterol increase, conjunctivitis, decubitus ulcer, drug eruption, peripheral edema, eosinophil count increase, fungal skin infection, KD, pharyngitis, renal tubular disorder, respiratory depression, skin erosion, skin injury, stomatitis, and urine positive for white blood cells. ADR, adverse drug reaction; AE, adverse event; anti-dsDNA, anti–double-stranded DNA; KD, Kawasaki disease; VGIH, polyethylene glycol-treated human immunoglobulin.

## Discussion

Infliximab achieved a greater defervescence rate within 48 h compared with VGIH in patients with IVIG-refractory KD. CALs occurred in one patient receiving infliximab (6.3%) and in three patients receiving VGIH (20.0%) up to day 21. No patient had a new CAL after day 21. Infliximab showed good tolerability.

Burns *et al*. reported that 11/12 (91.7%) patients who received infliximab had fever cessation within 24 h compared with 8/12 (66.7%) patients who received a second IVIG^14^. One reason why defervescence rates within 48 h in our trial were low compared with the Burns study might be that we defined the initial IVIG-refractory KD not only by duration of fever after initial IVIG infusion but also by the increase in inflammatory markers. Infliximab has been reported to achieve faster defervescence than an additional IVIG for patients with KD who were resistant to initial IVIG in the Korean randomized, controlled trial^16^. Also in the present trial, the earlier defervescence of infliximab compared with that of VGIH was shown by post-hoc analysis. The greater and earlier effect of infliximab on defervescence supports the potential usefulness of infliximab in patients with IVIG-refractory KD. The acute inflammatory markers reduced by infliximab treatment were similar to IVIG, even when patients were stratified by baseline levels of inflammatory markers (data not shown).

Prevention of CALs is one of the most important goals in KD treatment. Anti-TNF therapy directly improves endothelial cell function and also reduces inflammation^17^ and may, therefore, contribute to preventing the development of coronary artery abnormalities. A statistical difference in the incidence of CALs between infliximab and an additional IVIG was not observed in the Korean trial^16^. Our results were similar to that trial, however, we did not analyze the difference between two groups in consideration of sample size. In addition, our trial may be underpowered to detect the difference in CALs development, as patients with abnormal coronary arteries before enrollment were excluded. Incidences of CALs in the Korean trial and our trial were relatively low in the infliximab group (9.1% and 6.3%, respectively) versus the additional IVIG group (12.5% and 20.0%, respectively). Therefore, a large trial is necessary to conclude whether anti-TNF therapy may be more effective than additional IVIG for preventing the development of coronary artery abnormalities.

In a large phase 3 trial, Tremoulet *et al*. reported that infliximab treatment for IVIG-naïve patients with KD did not reduce treatment resistance, a primary endpoint; however, it reduced the number of days of fever^18^. Infliximab therapy was reported to decrease serum soluble TNF receptor 1 and IL-6 levels^19^ and to regulate the signaling pathways related to KD inflammation as well as IVIG resistance factors^20^. Therefore, infliximab may be more useful in IVIG-refractory KD via these mechanisms than in IVIG-naïve KD.

In our trial, infliximab was well tolerated, and no new safety concerns were observed. In the Tremoulet trial, infliximab was also reported as well tolerated and not associated with infusion reactions or infections^18^. Other studies also reported favorable safety profiles (no infusion reactions) for infliximab in patients with KD with persistent arthritis after IVIG or with IVIG-refractory KD^13,14,21^.

In the present trial, the increase in anti-dsDNA antibodies was observed in patients treated with infliximab similar to previous studies^22,23^. Although a link between these autoantibodies and lupus-like syndrome has been previously reported^24^, changes of anti-dsDNA antibodies in our trial were relatively transient and mostly anti-dsDNA IgM isotype, and lupus-like syndrome was not observed in any patient, as in previous reports^22,23^. Nevertheless, physicians should be aware of the risk of this complication. The reason for anti-dsDNA IgM increases in patients treated without infliximab in the VGIH group is unknown.

A previous trial reported peak serum concentrations of infliximab infusion similar to our findings^13^. The pharmacokinetics (PK) of infliximab in children with ulcerative colitis or Crohn’s disease^25,26^, who had already received infliximab in daily medical practice, were comparable with those in the present trial, supporting the good tolerability of infliximab.

Major limitations of this trial include the small sample size, the relatively short follow-up (56 days), and being an open-label trial. Larger and longer studies may help confirm the present results and longer-term outcomes (e.g. growth and development, the course of an aneurysm) with infliximab. In addition, the enrolled patients had “typical” KD with a uniform clinical presentation, but corticosteroid use was restricted, which may have affected the efficacy evaluation. Therefore, future studies should include younger patients and patients with atypical or incomplete KD.

Infliximab was associated with a higher defervescence rate within 48 h and more rapid resolution of fever after starting infusion compared with VGIH in patients with IVIG-refractory KD; this was potentially related to attenuated inflammation. Infliximab was well tolerated, and the rates of most AEs related to investigations or infections were similar in both groups. The results of this trial led to the approval of infliximab for the treatment of IVIG refractory KD in Japan in December 2015. As the infusion period for infliximab is much shorter than that of VGIH, this may reduce the amount of healthcare resources required during treatment. Infliximab, rather than a second dose of IVIG, may become a viable therapeutic option for patients with IVIG-refractory KD.

## Methods

### Study design and patients

This was a randomized, open-label, active-controlled, parallel-group, multicenter trial conducted at 10 medical institutions in Japan from May 2012 to September 2014 (NCT01596335; Trial registration date: May 9, 2012). The trial included patients diagnosed with KD: according to the Japanese diagnostic guidelines^27^; aged 1–10 years; refractory to initial IVIG therapy (2 g/kg), defined as persistent fever of ≥38.0 °C (axillary temperature) and ≥4 h within 24–36 h after completion of the initial IVIG infusion and increases in either white blood cell count, neutrophil count, or CRP within 24–36 h since before initial IVIG administration; fever of ≥37.5 °C at enrollment; and could be administered study drug within 8 days of illness onset (the first day). The main exclusion criteria were: patients with abnormal coronary arteries before enrollment (to assess effect on new CALs development); bacillus Calmette-Guérin vaccination within 6 months before enrollment or no bacillus Calmette-Guérin vaccination; treatments other than initial IVIG for KD; corticosteroids (prednisolone equivalent of ≥1 mg/kg/day) within 4 weeks before enrollment; history of infliximab and other biologics; abnormal laboratory results after consent; complication or a history of infections; history of hypersensitivity to IVIG; immunodeficiency; or serious complications requiring hospitalization. This trial was conducted in accordance with the ethical principles originating in the Declaration of Helsinki and in compliance with Good Clinical Practice and related regulations. The patients’ parents/legal guardians provided written informed consent. Prior to the conduct of the study, the protocol was reviewed and approved by each institutional review board of participating institution at Yokohama City University Hospital, Yokohama City University Medical Center, Yokosuka General Hospital Uwamachi, Hamamatsu University Hospital, National Hospital Organization Shikoku Medical Center for Children and Adults, Nagasaki University Hospital Medicine and Dentistry, Shinshu University School of Medicine, Kurashiki Central Hospital, Yamaguchi University Hospital, and Sasebo City General Hospital.

### Procedures

This trial included a screening period (from the time of obtaining written informed consent to the start of study drug administration) and an assessment period (from the start of study drug administration to day 56) (Supplementary Fig. S5). Patients were randomly assigned (1:1) to receive either a single dose of 5 mg/kg infliximab originator over 2 h or an additional single dose of 2 g/kg polyethylene glycol-treated human immunoglobulin (VGIH; Venoglobulin^®^ IH, Japan Blood Products Organization, Tokyo, Japan), a widely used IVIG in Japan, over 20 h via IV infusion according to label directions^28,29^ on day 0. Randomization was performed centrally using a dynamic allocation method, with sex and age as assignment factors. Because of the difference in their infusion periods, infliximab and VGIH were administered open-label. Concomitant use of systemic corticosteroids, immunomodulators (e.g. cyclosporine), and plasmapheresis were prohibited during the assessment period; however, these were permitted after evaluation at the time of discontinuation. The dose of acetylsalicylic acid ranged from 30–50 mg/kg/day for the acute stage and 3–5 mg/kg/day after defervescence; however, the dose could be changed for medical reasons, such as improvement of clinical symptoms of KD or the occurrence of AEs.

The primary endpoint was the defervescence rate within 48 h after the start of treatment with infliximab or VGIH. “Fever” was defined as a body temperature of ≥37.5 °C and “defervescence” as a lowering of body temperature to <37.5 °C for ≥48 h from the start of study drug administration. “Fever resolution” was defined as the time when the body temperature first dropped below 37.5 °C. Axillary body temperatures were measured every 4 h until defervescence, then once a day until day 56. Secondary endpoints included the overall defervescence rate until 48 h, duration of fever, incidence of CALs on days 21 and 56, Z-score until day 56, presence/absence of major symptoms (bilateral bulbar conjunctival congestion, lip/oral cavity changes, polymorphous rash, distal extremity changes, and non-suppurative cervical lymphadenopathy) in the acute stage, and laboratory variables (white blood cell count, neutrophil count, platelet count, albumin, and CRP) until day 56, which were previously investigated as possible predictors of CALs in IVIG-treated KD^30^. CALs were defined as a coronary artery with an internal diameter of ≥3 mm in patients aged <5 years or ≥4 mm in patients aged ≥5 years. CALs were assessed using echocardiography by a Central Review Committee, comprising three KD specialists who were blinded to patient information at all time points except baseline. Z-scores were determined using the lambda-mu-sigma method^31^. The PK endpoints included PK parameters derived from serum infliximab concentrations. Serum infliximab concentrations were measured at Mitsubishi Tanabe Pharma Corporation by enzyme-linked immunosorbent assay using anti-infliximab monoclonal antibodies from Janssen Biotech, Inc. (Horsham, PA, USA), with a detection limit of 0.1 μg/ml^32^. The safety endpoints were AEs and ADRs, as well as general laboratory tests during the study period. AEs were coded using MedDRA version 17.1. Antinuclear antibodies by Farr radioimmunoassay, anti-dsDNA IgG antibodies and anti-dsDNA IgG antibodies by enzyme-linked immunosorbent assay were measured at LSI Medience Corporation (Tokyo, Japan).

Although a Data and Safety Monitoring Board was not convened for this study, we had medical experts and a safety evaluation committee to review the data.

### Statistical analyses

The target sample size was determined to be 100 patients (50 per group), based on the incidence of IVIG-refractory KD in Japan (estimated at 1650 per year) and the possibility of conducting a study in this population. To estimate the power of this study, defervescence rate was estimated to be 80% in the infliximab group and 60% in VGIH group from the previous studies^15,33^, the non-inferiority margin was set at 10%, with a one-sided α level of 0.025 and 50 patients per group. In above assumptions, the power of non-inferiority test was calculated as 91%.

Analyses of primary endpoint and incidence of CALs were conducted in the full analysis set comprising all randomized patients who received at least one dose of the study drug and in whom the efficacy endpoints were measured at least once. Patients who discontinued the study prior to 48 h were defined as “non-responsive”. Other endpoints were calculated in patients from whom data were obtained at each time point. Safety analyses were conducted in the safety analysis set comprising all randomized patients who received at least one dose of the study drug and in whom safety data were collected at least once. The PK analysis set comprised patients who received infliximab and had at least one measurement of serum drug concentrations.

The primary endpoint was analyzed using a generalized linear model, with a likelihood ratio test and sex as a covariate. Defervescence rates within 48 h were shown as the least squares mean with 95% confidence interval. The defervescence rates by sex were shown as the proportion of patients who had fever cessation. Other secondary endpoints were analyzed using descriptive statistics. Kaplan–Meier plots were used to assess the overall defervescence rate, duration of fever, and the proportions of patients with major symptoms, excluding fever.

After consulting the Japanese Pharmaceutical and Medical Devices Agency, the study duration was extended and the sample size was reduced during the study, as patient recruitment was difficult owing to the very small number of refractory patients who met the eligibility criteria.

### Data Availability

The datasets generated during and/or analyzed during the current study are not publicly available but are available from the corresponding author on reasonable request.

## Electronic supplementary material

Supplementary Information
