# Supplementary material for: Infliximab versus intravenous immunoglobulin for refractory Kawasaki disease: a phase 3, randomized, open-label, active-controlled, parallel-group, multicenter trial
Source: Sci Rep. 2018 Jan 31;8:1994. doi: 10.1038/s41598-017-18387-7 (PMC5792468; doi:10.1038/s41598-017-18387-7)
Supplement: Supplementary file 1 — Supplementary Information [file 41598_2017_18387_MOESM1_ESM.pdf]

SUPPLEMENTARY INFORMATION

Infliximab versus intravenous immunoglobulin for refractory Kawasaki disease: a phase 3, randomized, open-label, active-controlled, parallel-group, multicenter trial

Masaaki Mori[1,15], Takuma Hara[1,16], Masako Kikuchi,[2] Hiroyuki Shimizu[3], Tomoyuki Miyamoto[4], Satoru Iwashima[5,17], Tatsuya Oonishi[6], Kunio Hashimoto[7], Norimoto Kobayashi[8], Kenji Waki[9], Yasuo Suzuki[10], Yoshikazu Otsubo[11], Hiroshi Yamada[12], Chikao Ishikawa[12], Taichi Kato[13] & Shigeto Fuse[14]

[1]Yokohama City University Medical Center, Yokohama, Japan.

[2]Department of Pediatrics, Yokohama City University Hospital, Yokohama, Japan.

[3]Children's Medical Center, Yokohama City University Medical Center, Yokohama, Japan.

[4]Department of Pediatrics, Yokosuka General Hospital Uwamachi, Yokosuka, Japan.

[5]Hamamatsu University School of Medicine, Hamamatsu, Japan.

[6]Department of Pediatrics, National Hospital Organization Shikoku Medical Center for Children and Adults, Zentsuji, Japan.

[7]Department of Pediatrics, Nagasaki University Graduate School of Biomedical Sciences, Nagasaki, Japan.

[8]Department of Pediatrics, Shinsyu University School of Medicine, Matsumoto, Japan.

[9]Department of Pediatrics, Kurashiki Central Hospital, Kurashiki, Japan.

[10]Department of Pediatrics, Yamaguchi University Graduate School of Medicine, Ube, Japan.

[11]Department of Pediatrics, Sasebo City General Hospital, Sasebo, Japan.

[12]Mitsubishi Tanabe Pharma Corporation, Tokyo, Japan.

[13]Department of Pediatrics, Nagoya University Hospital, Nagoya, Japan.

[14]Department of Pediatrics, NTT Sapporo Medical Center, Sapporo, Japan.

[15]Present address: Department of Lifetime Clinical Immunology, Tokyo Medical and Dental University, Tokyo, Japan.

[16]Present address: Department of Pediatrics, Hara Children's Clinic, Tokorozawa, Japan.

[17]Present address: Department of Pediatrics, Chutoen General Medical Center, Kakegawa, Japan.

Supplementary Fig. S1 Change in body temperature over time

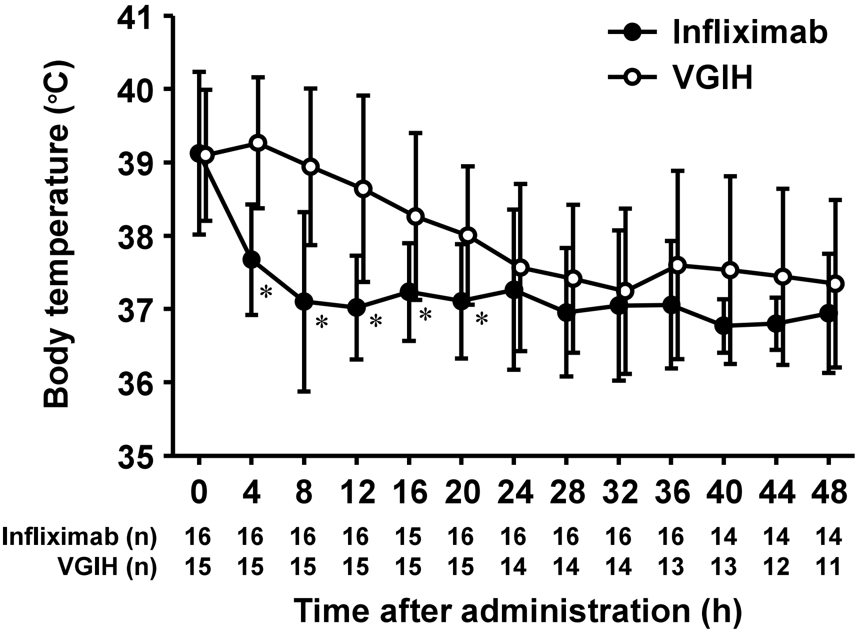

Data are shown as mean (SD). *p < 0.01 (unpaired t-test). SD, standard deviation; VGIH, polyethylene glycol-treated human immunoglobulin.

Supplementary Fig. S2 Effect on Z_{max} (largest of the right coronary artery, left main coronary artery, left anterior descending artery, and left circumflex coronary artery internal diameters) at days 0, 3, 7, 14, 21, and 56 in the patients who were evaluated coronary artery internal diameters (Z-score) after the start of treatment

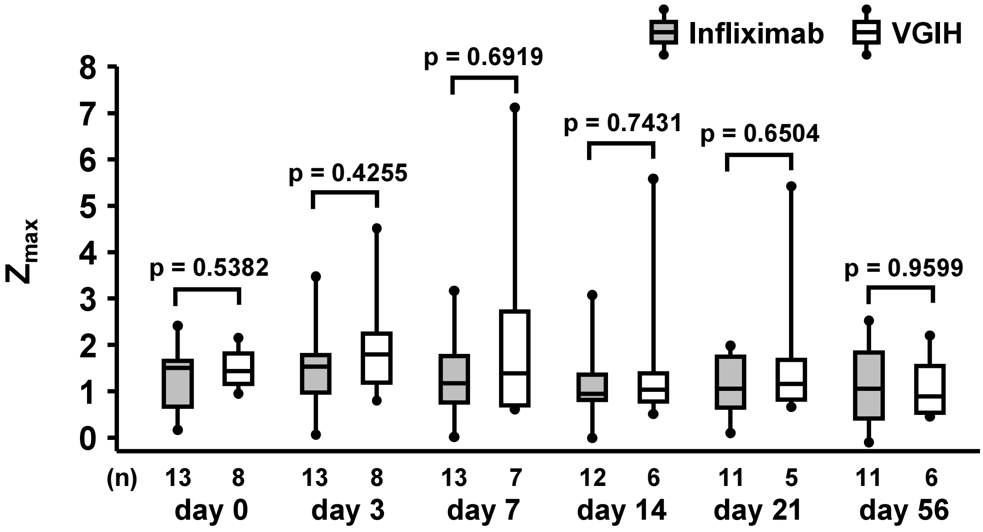

Results are presented in boxplot graphs. P values were calculated with a Mann-Whitney U test.

VGIH, polyethylene glycol-treated human immunoglobulin.

Supplementary Fig. S3 Changes over time in the proportion of patients with major symptoms (excluding fever) in the acute stage

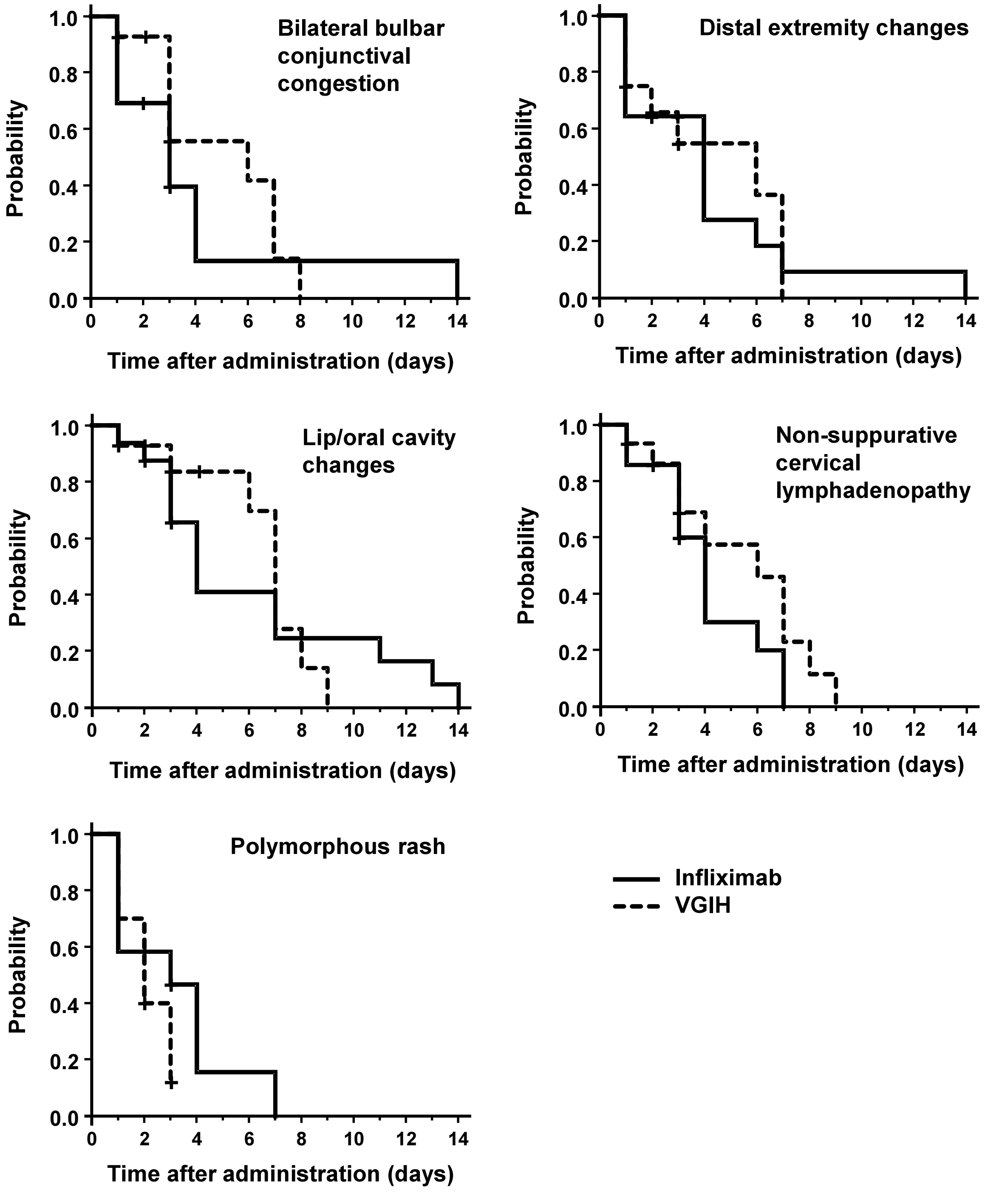

VGIH, polyethylene glycol-treated human immunoglobulin.

Supplementary Fig. S4 Changes in inflammation-related laboratory variables over time

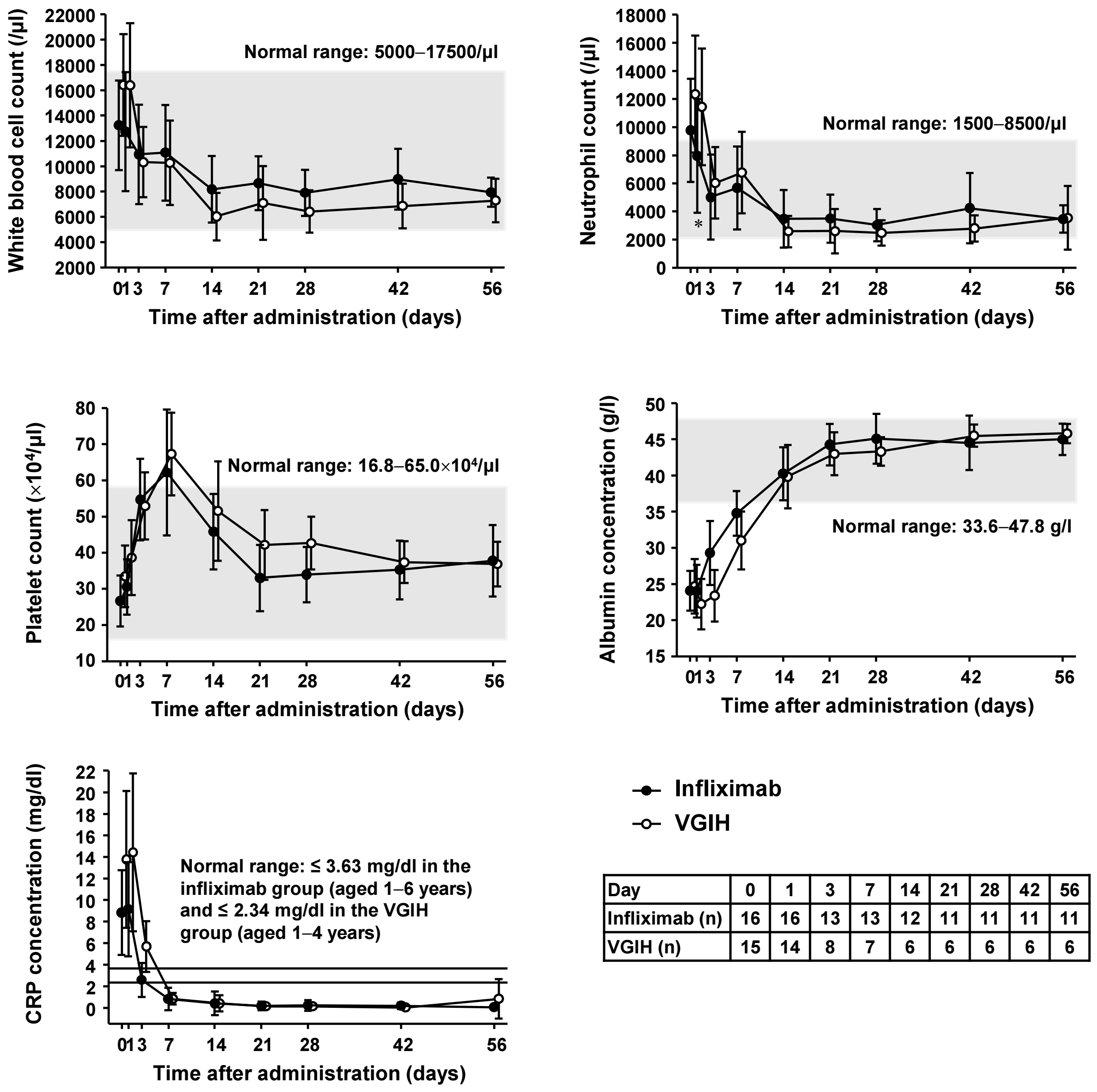

Day	0	1	3	7	14	21	28	42	56
Infliximab (n)	16	16	13	13	12	11	11	11	11
VGIH (n)	15	14	8	7	6	6	6	6	6

Values are presented as the mean (SD). Normal ranges of white blood cell count, neutrophil count, platelet count, albumin, and CRP were 5000-17,500/µl, 1500-8500/µl, 16.8-65.0 × 10^4/µl, 33.6-47.8 g/l, and ≤ 3.63 mg/dl in the infliximab group (aged 1-6 years) and ≤ 2.34 mg/dl in the VGIH group (aged 1-4 years). Other endpoints included body temperatures on days 0-48. CRP, C-reactive protein; SD, standard deviation; VGIH, polyethylene glycol-treated human immunoglobulin.

*n=15.

Supplementary Fig. S5 Study design

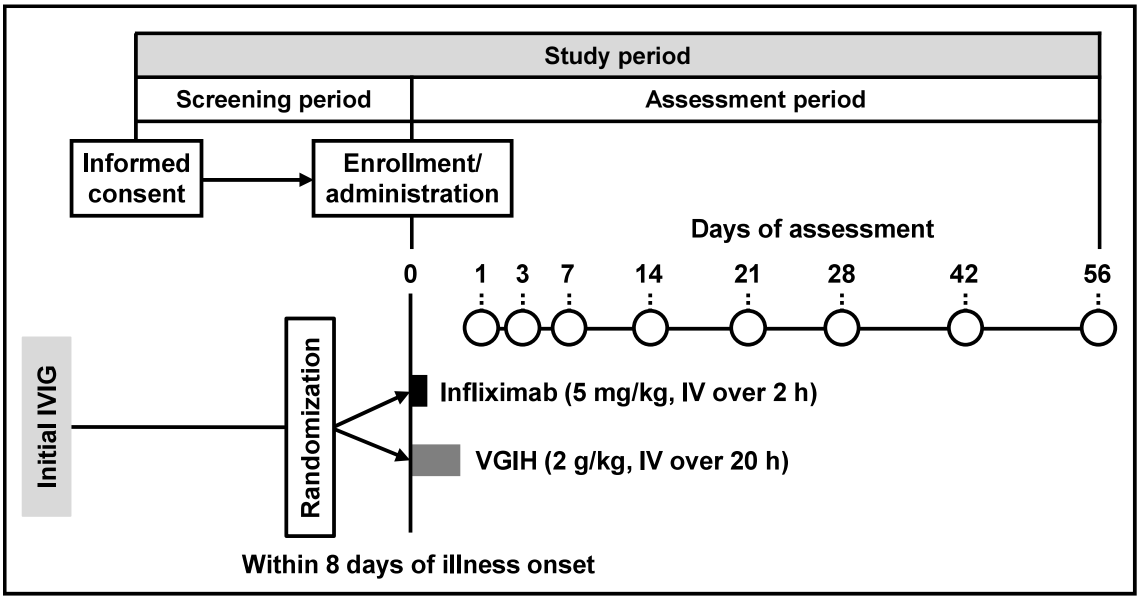

The following assessments were carried out after a single dose of infliximab or VGIH in patients with KD refractory to initial IVIG.

Patients had received one of four approved IVIG formulations in Japan as initial IVIG therapy: VGIH (Venoglobulin® IH, Japan Blood Products Organization, Tokyo, Japan), freeze-dried sulfonated human normal immunoglobulin (Kenketsu Venilon®-I, Teijin Co., Ltd, Tokyo, Japan), freeze-dried VGIH (Kenketsu Glovenin®-I, Nihon Pharmaceutical Co., Ltd, Tokyo, Japan), or pH4-treated acidic human normal immunoglobulin (Nisseki Polyglobin®-N 5%, 10% I.V., Japan Red Cross Society, Tokyo, Japan). IV, intravenous; IVIG, intravenous immunoglobulin; KD, Kawasaki disease; VGIH, polyethylene glycol-treated human immunoglobulin.
